# Association of Cardiac Electrical Disorders With KCND3 Gene Mutation

**DOI:** 10.7759/cureus.34597

**Published:** 2023-02-03

**Authors:** Md Ripon Ahammed, Fariha Noor Ananya

**Affiliations:** 1 Internal Medicine, Icahn School of Medicine at Mount Sinai, Queens Hospital Center, New York, USA; 2 Internal Medicine, Dhaka Medical College and Hospital, Dhaka, BGD

**Keywords:** sudden cardiac death, atrial fibrillation, brugada syndrome, early repolarization syndrome, cardiac electrical disorder, heart, kcnd3

## Abstract

Globally, cardiac channelopathies leading to electrical disorders are responsible for a significant number of sudden cardiac deaths without structural heart disease. Many genes encoding different ion channels in the heart were identified and their impairment was found to be associated with life-threatening cardiac abnormalities. KCND3, one of the genes expressed both in the heart and brain, is reported to have an association with Brugada syndrome, early-onset atrial fibrillation, early repolarization syndrome, and sudden unexplained death syndrome. KCND3 genetic screening could be a promising tool for functional studies for an understanding of the pathogenesis and genetic determinants of the above-mentioned electrical disorders.

## Introduction and background

The incidence of sudden cardiac death (SCD) is around 0.3 million deaths annually [1], and in young people (<35 years old), it is around 1.3 to 3.2 per 0.1 million person-years [2]. Around 40% of the young cases and 5% to 10% of total SCDs were found to have a normal heart structure in an autopsy, indicating possible cardiac channelopathies leading to an electrical disorder [1,2]. Many of them are due to heritable defects in the function of cardiac ion channels [1]. A significant portion of the identified genes till now related to electrical disturbances encode the important ion channel pore-forming units or associated proteins that regulate ion channels or interact with them [2]. KCND3 (potassium voltage-gated channel subfamily D member 3) is one of the genes that code Kv4.3 formulating transient outward potassium currents (Ito) and is found in both the cardiac and cerebral tissues. Mutations of KCND3 have been illustrated to be culpable for the channelopathies including early repolarization syndrome (ERS), Brugada syndrome (BrS), atrial fibrillation (AF) of early-onset, sudden unexplained cardiac death syndrome with negative autopsy [3]. This review article attempts to briefly lay out the relationship of the KCND3 gene with different cardiac channelopathies. We aim to briefly address the pathophysiological and genetic mechanisms including the potential clinical aspects such as the preventive approach.

## Review

KCND3 in the heart and its role in cardiac channels

Cardiac ion channels are important membrane proteins, encoded by different genes, consisting of pore-forming, alpha subunits mediating ion currents, and regulatory beta subunits [4]. A complex balance of serial activation and inactivation of these ionic channels, ingoing depolarizing currents (Na+ ion and Ca2+ ion) and outgoing repolarizing currents (K+ ion), results in action potential generation [4,5]. Any disruption of this balance due to defective ion channels (as a result of genetic mutation), leads to a potential risk for unstable electrical activity or cardiac arrhythmia [4].

KCND3 gene encodes Kv4.3, which is the alpha subunit of voltage-gated fast transient outward potassium channel (Ito), found in heart and brain tissues [6]. In the heart, the right ventricle has the highest KCND3 expression [1]. Several KCND3 mutations of autosomal dominant inheritance were reported associated with Brugada syndrome (BrS) and spinocerebellar ataxia (SCA) [7]. Multiple de novo mutations were reported with different cardiac electrical disorders [3,7,8]. Functional gain and loss of the potassium ion channel coded by KCND3 can cause cardiac channelopathies (Brugada syndrome, early-onset atrial fibrillation, early repolarization syndrome) and spinocerebellar ataxia subsequently [1,6,9]. At the ionic and cellular levels, inadequate sodium (INa) or calcium (ICa) depolarizing ingoing current coupling with a transmural gradient ( endocardium < epicardium) of the right ventricle (RV) created by KCND3-dependent Kv4.3 alpha subunit is hypothesized to cause a shift of the current towards outward, dome loss of action potential, the elevation of ST segment on electrocardiogram, phase-II re-entry-induced local re-excitation, and the origination of multifocal ventricular tachycardia (VT) or fibrillation of ventricle [1].

Early repolarization syndrome (ERS)

Impaired cardiac ion channels, including transient outward potassium current (Ito), can cause early repolarization patterns (ERP) in electrocardiogram (ECG) [10]. It is evidenced by at least 1 mm elevation of J point (QRS-ST junction) in ≥2 adjacent inferior and/or lateral leads. ERP prevalence in the general population is around 2-13% and is more common in younger athletic males [11]. It was considered to be a benign ECG phenotype, however, in a multi-center study, Haïssaguerre et al. illustrated its strong association with an elevated risk of ventricular fibrillation (VF) as well as sudden cardiac death (SCD), i.e., early repolarization syndrome (ERS) [11,12]. Teumer et al. found a significant genome-wide locus in the KCND3 gene was able to be replicated successfully in 1,124 cases of ERP by identifying rs1545300 as the predominant single nucleotide polymorphisms (SNP) at the KCND3 locus following combined replication cohorts and meta-analysis. This not only provided insights into genetic determinants for ERP but also into pathophysiological mechanisms [11].

Though cardiocerebral channelopathy associated with ERS is quite uncommon [6], few cases have been reported that describe this association where KCND3 mutation is found to be responsible for these phenomena [3,6,8,10,11,13,14]. The ECG of an 11-year-old girl showed ERP; she was known to have febrile seizures since age two and intellectual disability was found at age six [6]. A mutation in KCND3 G306S (c.916G > A) and chr-1-112524433 was found in this individual by whole-exome sequencing without any significant family history, thus confirmed de novo by Sanger sequencing [6]. ERS was found to be linked with epilepsy and intellectual disability in another reported case of a teenage girl, whose sudden death at 16 led to the diagnosis of ERS, a de novo KCND3 V392I mutation was found in her mother, this mutation was also found in her 19-year-old sister who was found to be suffering from ERS as well [3]. ERS associated with epilepsy and AF has been reported in another six-year-old patient who was found to have a missense mutation in the KCND3 gene [13], making the association between cardiocerebral channelopathy and ERS evident in these individuals. Takayama et al. illustrated a de novo KCND3 Gly306Ala (c.917g>c) heterozygous mutation in a 12-year-old boy whose ECG shows elevation of J points in multiple leads [8]. Increased expression of mutant Ito channels in this individual is suggested to be the underlying pathology of ERS [8]. Chauveau et al. reported an intermittent ERP and non-fatal cardiac arrest in a patient who was found to have an entire KCND3 gene duplication detected by next-generation sequencing (NGS). KCND3 gene duplication is suggested to cause increased transmural Ito density leading to ventricular arrhythmia that was seen in this 26-year-old young man [15].

Brugada syndrome (BrS)

Brugada syndrome (BrS) is a cardiac channelopathy related to an elevated risk of SCD, characterized by the presence of a specific pattern of electrocardiographic findings of right bundle branch block (RBBB) along with a constant elevation of the ST-segment in right precordial leads [5]. There were three repolarization patterns described: 1. Type-1 pattern with a ≥ 2 mm coved ST-segment elevation with a downward T-wave afterward, features being present in more than one chest lead (V1 to V3); Type 2 pattern with an ST-segment elevation with a saddle-shape appearance with a subsequent upward or biphasic T-wave; Type 3 pattern with a ≤ 1 mm ST-segment elevation in V1 to V3 leads, which is either coved-type or saddle-back shaped [5]. It is hypothesized that conduction slowing of the right ventricular outflow (RV) tract or transmural voltage gradients between the right ventricular epicardium and myocardium as a result of disequilibrium between INa and Ito channels result in the dispersion of repolarization cause the typical ST-segment elevation pattern. A high number of genetic mutations have been discovered related to cardiac channelopathies [5]. BrS was found to be associated with >500 pathogenic variations, which support an autosomal dominant (AD) inheritance pattern [16]. Around 35% of patients with BrS have a genetic cause where sodium channel protein type-5 subunit alpha (SCN5A) mutation is the most prevalent one [5]. Abnormality in the Kv4.3 potassium channel encoded by the KCND3 gene has also been reported in BrS [17]. The mutations causing gain of function in KCND3 (L450F and G600R) were described in patients of BrS with elevation ST of the segment in the precordial leads V1 to V3 as a result of rising peak Ito current density [9,11,18]. Li et al. reported a heterozygous variant of the novel KCND3, Arg431His, in one sporadic case of BrS [17]. Dehghani-Samani et al. illustrated the gain of function of the gene, L450F & G600R, resulting in characteristic ECG findings in V1-V3 because of increasing peak Ito current density [9]. Li et al. illustrated that genetic screening in the clinic for KCND3 could be useful for a comprehension of the pathogenesis of BrS and illustrating useful risk stratification for patients in outpatient settings [17].

Atrial fibrillation (AF)

AF is the most frequently seen persistent heart arrhythmia that leads to several complications and is associated with poor disease outcomes [19-21]. The prevalence of AF globally was reported as around 46.3 million people in 2016 and the lifetime risk of developing AF was one out of every three white individuals and one out of every five black individuals [22]. Olesen et al. reported that a mutation causing a gain of function (A545P) in Kv4.3 caused a shortening of the action potential of the atrium leading to AF without any structural heart diseases or obvious risk factors in a young patient [18]. Another study reported two teenage siblings having paroxysmal atrial fibrillation that is associated with a V392I mutation in the KCND3 gene confirmed by Sanger sequencing [3]. A genomic study of their parents revealed de novo KCND3 V392I mutation in their mother only, who suffered two syncopal episodes in the past on two different occasions and her 12 lead ECG showed a brief period of PAF with no symptoms [3]. PAF associated with ERS and epilepsy was found in a six-year-old girl whose genetic study revealed a missense mutation in the KCND3 gene [13]. Mutation in 916 G>A (G306S) KCND3 has been reported in another eight-year-old patient having atrial fibrillation, epilepsy, and developmental delay [23]. Huang et al. reported a novel T361S mutation in a young patient having lone AF [7]. Electrophysiological studies showed that a T361S mutation resulted in a gain of function of the Ito channel, leading to increased expression of pore-forming Kv4.3, and atrial action potential shortening causing lone AF in these individuals [7].

Sudden unexplained death syndrome (SUDS)

Sudden unexplained death syndrome (SUDS) is defined as an unanticipated death naturally in healthy young people, where any life-threatening disease cannot be identified as an explanation of death’s etiology. Many studies reported the association of SUDS with genetically modified ion channels of the heart [24]. Giudicessi et al. reported an association of the KCND3 mutation with one case of sudden infant death syndrome (SIDS) and two cases of sudden unexplained death syndrome (SUDS) [25]. Two novel mutations p.Gly600Arg and p.Val392Ile were found to be associated with SUDS. Heterogeneous mutation p.Ser530Pro was found to be SIDS-related and reflected a wild-type phenotype electrophysiologically [25].

Cardiocerebral channelopathy

Kv4.3 protein, the alpha subunit of the fast transient outward potassium (Ito) channel encoded by the KCND3 gene is expressed both in heart and brain tissues. Gain-of-function of the Ito channel caused by mutated KCND3 leads to BrS, AF, and ERS, and on the other hand, loss-of-function mutation of the channel causes spinocerebellar ataxia [6]. Nakajima et al. reported KCND3 V392I mutation-induced cardiocerebral channelopathy in two siblings displaying both cerebral (intellectual disability and epilepsy) and cardiac (paroxysmal AF and ERS) phenotypes [3]. Ali et al. identified a case of KCND3 mutation that led to simultaneous epilepsy, developmental delay, and atrial fibrillation [23]. A novel KCND3 c.1054A>G (p.T352A) variant was reported in a patient with cerebellar ataxia from a Korean family [26]. Zhang et al. reported KCND3 mutation-induced cardiocerebral channelopathy with epilepsy and intellectual disability as the cerebral phenotype and ERS and AF as the cardiac phenotype [6].

Therapeutics

Ye et al. reported a KCND3-associated potent transient outward current blocker named Acacetin which has the potentiality for being a novel therapeutic agent for Kv4.3 gain-of-function-related EPS. In this preclinical study, the pathogenic KCND3 mutation p.Val392Ile (associated with increased peak Ito current) was generated by site-directed mutagenesis engineering. It was found that Acacetin significantly inhibited KCND3 p.Val392Ile-associated peak Ito current leading to the abolishment of the accentuated action potential [27]. In a similar study, Ye et al. demonstrated that Acacetin was successful in dramatically inhibiting KCND3-L450F (gain of function leading to increased peak Ito density) responsible for BrS [28]. However, further research is required in this potential area.

A summary of the studies included in our review is shown in Table 1.

**Table 1:** List of studies demonstrating the relationship between KCND3 and different cardiac channelopathies

Author	Year of publication	Type of study	Relevant Findings
Zhang et al. [6]	2022	Case Report	KCND3 mutation induced cardiocerebral channelopathy with early repolarization syndrome (ERS) and atrial fibrillation (AF)n as cardiac phenotype.
Choubey et al. [13]	2022	Case report	KCND3 missense mutation association with AF with ERS.
Ye et al. [27]	2022	Original article	KCND3 V392I mutation was reported in an 18-year-old young man with ERS. Acacetin, a potent Ito current blocker, was studied as a novel therapeutic agent for KCND3-induced ERS. Acacetin significantly inhibited 93.2% of KCND3 V392I peak Ito current.
Ali et al. [23]	2022	Case report	KCND3 mutation caused simultaneous epilepsy, developmental delay, and atrial fibrillation.
Nakajima et al. [3]	2020	Case Report	KCND3 V392I mutation induced cardiocerebral channelopathy in two siblings displaying both cerebral ( intellectual disability & epilepsy) and cardiac (paroxysmal AF and ERS) phenotypes.
Li et al. [17]	2020	Original study	A novel heterozygous variant of KCND3 c.1292G>A (R431H, Arg431His) was found in the pathogenesis of BrS.
Teumer et al. [11]	2019	Meta-Analysis	A locus in the KCND3 gene is strongly associated with ERS.
Takayama et al. [8]	2019	Experimental study	A novel KCND3 Gly306Ala mutation was identified in association with ERS.
Yao et al. [10]	2018	Original study	Coexpression of Sodium channel subunit beta-1 (SCN1Bβ) and wild-type KCND3 caused an increase in the transient outward potassium current (Ito).
Portero et al. [29]	2018	Original article	KCND3 gain-of-function mutation was found in association with BrS.
Huang et al. [7]	2017	Original study	A novel KCND3 T361S missense mutation was identified in a Chinese patient with AF.
Verweij et al. [30]	2014	Original study	Single-nucleotide polymorphism (SNP) in KCND3 was associated with P wave duration.
Olesen et al. [18]	2013	Original study	A novel KCND3 A545P mutation was identified in lone AF.
Giudicessi et al. [25]	2012	Original study	Sudden unexplained death syndrome (SUDS)-related KCND3 p.Gly600Arg and p.Val392Ile were identified.
Delpón et al. [31]	2008	Comparative study	Co-transfection of KCNE3 with KCND3 caused an increase in the Ito intensity significantly. Ito plays an important role in the expression of Brugada syndrome.
Raudenská et al. [32]	2008	Original study	Genetic analysis was done in 12 cases of long QT syndrome, and no mutation related to KCND3 was found.

## Conclusions

In summary, the KCND3 gene is associated with different cardiac channelopathies, mainly Brugada syndrome, early-onset atrial fibrillation, early repolarization syndrome, and sudden unexplained death syndrome. The KCND3 genetic screening could be a promising tool for the comprehension of pathogenesis and genetic determinants of these electrical disorders. Further research is required to establish KCND3 as a screening tool for the detection of potential cardiac abnormality and take necessary steps to prevent the burden of sudden cardiac death.
